# *catena*-Poly[[(5-bromopyridine-3-carbox­yl­ato)dimetyltin(IV)]-μ-5-bromopyridine-3-carboxyl­ato]

**DOI:** 10.1107/S1600536807067165

**Published:** 2007-12-21

**Authors:** Zhongjun Gao

**Affiliations:** a Department of Chemistry, Jining University, Shandong 273155, People’s Republic of China

## Abstract

The title compound, [Sn(CH_3_)_2_(C_6_H_3_BrNO_2_)_2_], possesses an infinite chain structure owing to the presence of Sn—N bridges between adjacent mol­ecules. The SnO_4_NC_2_ centre has a distorted penta­gonal–bipyramidal geometry with the C atoms in the axial positions.

## Related literature

For related literature, see: Tiekink (1991 ▶); Yin *et al.* (2006 ▶).
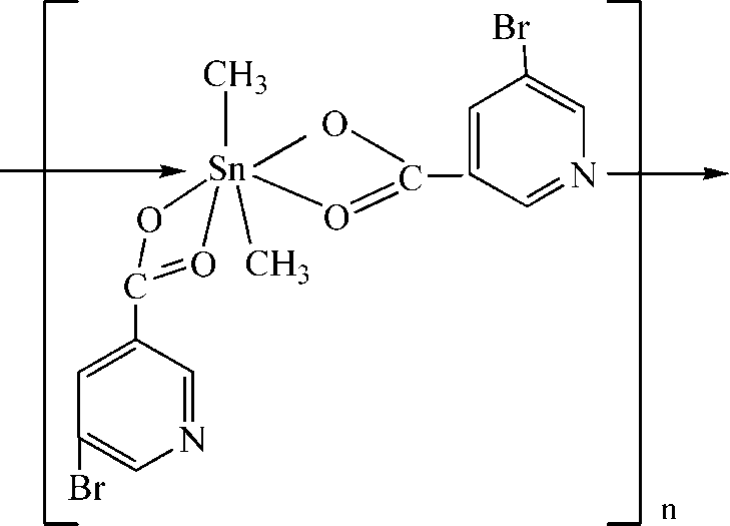


## Experimental

### 

#### Crystal data

[Sn(CH_3_)_2_(C_6_H_3_BrNO_2_)_2_]
*M* *_r_* = 550.77
Triclinic, 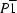

*a* = 7.579 (4) Å
*b* = 8.212 (5) Å
*c* = 14.894 (8) Å
α = 74.962 (7)°
β = 77.733 (8)°
γ = 88.642 (8)°
*V* = 874.4 (8) Å^3^
*Z* = 2
Mo *K*α radiation
μ = 6.05 mm^−1^
*T* = 298 (2) K
0.27 × 0.12 × 0.02 mm

#### Data collection

Siemens SMART CCD diffractometer
Absorption correction: multi-scan (*SADABS*; Sheldrick, 1996 ▶) *T*_min_ = 0.292, *T*_max_ = 0.889
4540 measured reflections
3165 independent reflections
2652 reflections with *I* > 2σ(*I*)
*R*_int_ = 0.029

#### Refinement

*R*[*F* ^2^ > 2σ(*F* ^2^)] = 0.060
*wR*(*F* ^2^) = 0.162
*S* = 1.04
3165 reflections
210 parameters
H-atom parameters constrained
Δρ_max_ = 3.01 e Å^−3^
Δρ_min_ = −1.02 e Å^−3^

### 

Data collection: *SMART* (Siemens, 1996 ▶); cell refinement: *SAINT* (Siemens, 1996 ▶); data reduction: *SAINT*; program(s) used to solve structure: *SHELXS97* (Sheldrick, 1997*a* ▶); program(s) used to refine structure: *SHELXL97* (Sheldrick, 1997*a* ▶); molecular graphics: *SHELXTL* (Sheldrick, 1997*b* ▶); software used to prepare material for publication: *SHELXTL*.

## Supplementary Material

Crystal structure: contains datablocks I, global. DOI: 10.1107/S1600536807067165/hb2677sup1.cif

Structure factors: contains datablocks I. DOI: 10.1107/S1600536807067165/hb2677Isup2.hkl

Additional supplementary materials:  crystallographic information; 3D view; checkCIF report

## Figures and Tables

**Table 1:** Selected bond lengths (Å)

Sn1—C13	2.089 (8)
Sn1—C14	2.086 (8)
Sn1—O1	2.189 (5)
Sn1—O2	2.546 (6)
Sn1—O3	2.482 (6)
Sn1—O4	2.175 (5)
Sn1—N1^i^	2.710 (6)

Symmetry code: (i) 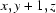.

**Fig. 1.**
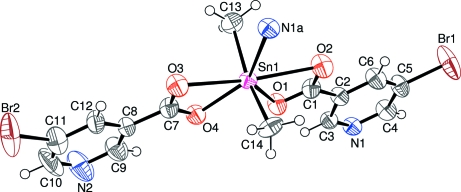
The molecular structure of (I) showing 50% displacement ellipsoids (arbitrary spheres for the H atoms). N1a is at the symmetry position (x, y + 1, z).

**Fig. 2.**
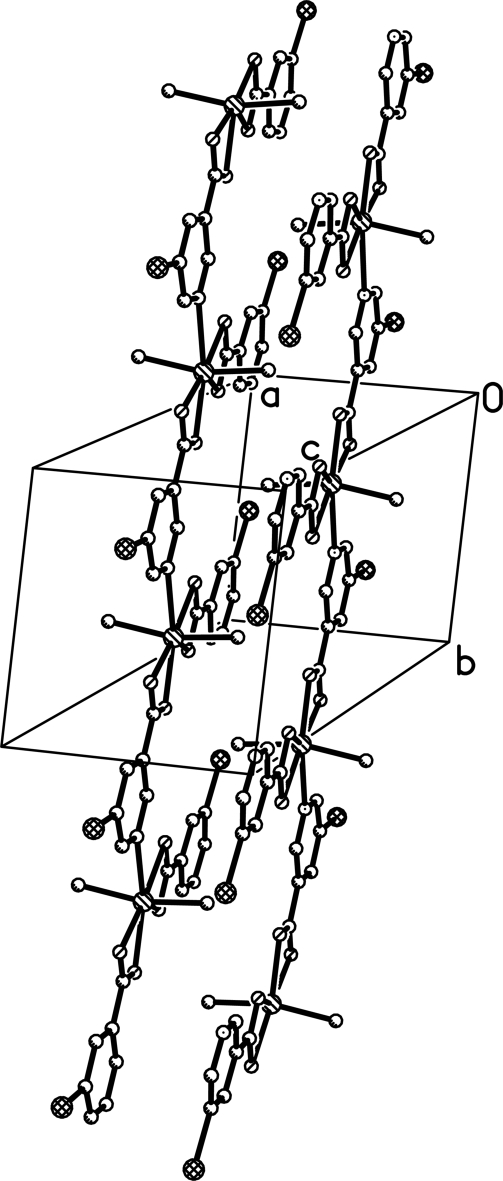
Polymeric chain formation in (I). H atoms omitted for clarity.

## supplementary crystallographic information

### Comment

The title compound, (I), possesses an infinite one-dimensional chain structure arising from Sn—N bridges (Fig. 1 and Table 1). As shown in Fig. 2, both carboxylate ligands chelate the Sn atom *via* the O atoms. One of the ligands also bridges a translationally related Sn atom *via* the N1 atom to form [010] chains.

The overall configuration at tin atom is best decribed as distorted pentagonal geometry with the C13 and C14 in the apical positions [C13—Sn1—C14 = 161.4 (4)°]. The sum of the equatorial angles about tin is 360°, indicating approximate co-planarity for these atoms. While the SnO_4_NC_2_ coordination geometry in (I) is similar to that seen recently in the structure of dioctyltin(IV) bis(2-pyrazinecarboxylate) (Yin *et al.*, 2006), this type of coordination is, in general, rare in this class of compound (Tiekink, 1991).

### Experimental

A mixture of dimetyltin oxide (0.329 g, 2.0 mmol) and 5-bromo-nicotinic acid (0.808 g, 4.0 mmol), in methanol (50 ml) was heated under reflux for 5 h. The clear solution was evaporated under vacuum. The product was crystallized from a mixture of dichloromethane/ethanol (1:1) to yield colourless plates of (I). Yield 0.860 g, 78%, m.p. 422 K. Analysis, calculated for C_14_H_12_Br_2_N_2_O_4_Sn: C 30.53, H 2.20, N 5.09%; found: C 30.56, H 2.15, N 5.13%.

### Refinement

The H atoms were included in the riding model approximation with C—H 0.93–0.96 Å, and *U*_iso_(H) = 1.2*U*_eq_(C) or 1.5*U*_eq_(methyl C).

### Crystal data

**Table:**

[Sn(CH_3_)_2_(C_6_H_3_BrNO_2_)_2_]	*Z* = 2
*M**_r_* = 550.77	*F*_000_ = 524
Triclinic, *P*1	*D*_x_ = 2.092 Mg m^−^^3^
Hall symbol: -P 1	Mo *K*α radiation λ = 0.71073 Å
*a* = 7.579 (4) Å	Cell parameters from 1572 reflections
*b* = 8.212 (5) Å	θ = 2.6–25.2º
*c* = 14.894 (8) Å	µ = 6.05 mm^−^^1^
α = 74.962 (7)º	*T* = 298 (2) K
β = 77.733 (8)º	Plate, colourless
γ = 88.642 (8)º	0.27 × 0.12 × 0.02 mm
*V* = 874.4 (8) Å^3^	

### Data collection

**Table:**

Siemens SMART CCD diffractometer	3165 independent reflections
Radiation source: fine-focus sealed tube	2652 reflections with *I* > 2σ(*I*)
Monochromator: graphite	*R*_int_ = 0.029
*T* = 298(2) K	θ_max_ = 25.5º
ω scans	θ_min_ = 2.6º
Absorption correction: multi-scan(SADABS; Sheldrick, 1996)	*h* = −8→9
*T*_min_ = 0.292, *T*_max_ = 0.889	*k* = −9→9
4540 measured reflections	*l* = −15→18

### Refinement

**Table:**

Refinement on *F*^2^	Secondary atom site location: difference Fourier map
Least-squares matrix: full	Hydrogen site location: inferred from neighbouring sites
*R*[*F*^2^ > 2σ(*F*^2^)] = 0.060	H-atom parameters constrained
*wR*(*F*^2^) = 0.162	*w* = 1/[σ^2^(*F*_o_^2^) + (0.1072*P*)^2^] where *P* = (*F*_o_^2^ + 2*F*_c_^2^)/3
*S* = 1.04	(Δ/σ)_max_ < 0.001
3165 reflections	Δρ_max_ = 3.01 e Å^−^^3^
210 parameters	Δρ_min_ = −1.02 e Å^−^^3^
Primary atom site location: structure-invariant direct methods	Extinction correction: none

### Special details

**Table:**

Geometry. All e.s.d.'s (except the e.s.d. in the dihedral angle between two l.s. planes) are estimated using the full covariance matrix. The cell e.s.d.'s are taken into account individually in the estimation of e.s.d.'s in distances, angles and torsion angles; correlations between e.s.d.'s in cell parameters are only used when they are defined by crystal symmetry. An approximate (isotropic) treatment of cell e.s.d.'s is used for estimating e.s.d.'s involving l.s. planes.
Refinement. Refinement of *F*^2^ against ALL reflections. The weighted *R*-factor *wR* and goodness of fit *S* are based on *F*^2^, conventional *R*-factors *R* are based on *F*, with *F* set to zero for negative *F*^2^. The threshold expression of *F*^2^ > σ(*F*^2^) is used only for calculating *R*-factors(gt) *etc*. and is not relevant to the choice of reflections for refinement. *R*-factors based on *F*^2^ are statistically about twice as large as those based on *F*, and *R*- factors based on ALL data will be even larger.

### Fractional atomic coordinates and isotropic or equivalent isotropic displacement parameters (Å^2^)

**Table:**

	*x*	*y*	*z*	*U*_iso_*/*U*_eq_	
Sn1	0.27918 (6)	0.22587 (5)	0.41226 (3)	0.0314 (2)	
N1	0.3215 (9)	−0.4573 (7)	0.2957 (5)	0.0385 (14)	
N2	0.1125 (16)	0.0212 (13)	0.8575 (6)	0.085 (3)	
O1	0.2868 (8)	−0.0342 (6)	0.4001 (4)	0.0437 (13)	
O2	0.3393 (9)	0.1381 (6)	0.2569 (4)	0.0515 (15)	
O3	0.2327 (8)	0.3727 (6)	0.5412 (4)	0.0471 (14)	
O4	0.2194 (7)	0.0988 (6)	0.5634 (4)	0.0414 (12)	
C1	0.3192 (11)	−0.0074 (9)	0.3115 (6)	0.0389 (17)	
C2	0.3315 (10)	−0.1571 (9)	0.2713 (5)	0.0358 (16)	
C3	0.3136 (10)	−0.3225 (9)	0.3298 (6)	0.0371 (16)	
H3	0.2955	−0.3384	0.3953	0.045*	
C4	0.3463 (12)	−0.4365 (10)	0.2031 (6)	0.048 (2)	
H4	0.3519	−0.5314	0.1795	0.057*	
C5	0.3641 (12)	−0.2788 (10)	0.1398 (6)	0.047 (2)	
C6	0.3554 (13)	−0.1378 (10)	0.1752 (6)	0.048 (2)	
H6	0.3657	−0.0303	0.1339	0.058*	
C7	0.2118 (11)	0.2291 (10)	0.5950 (6)	0.0393 (17)	
C8	0.1759 (11)	0.2050 (10)	0.7006 (5)	0.0388 (17)	
C9	0.1421 (14)	0.0468 (12)	0.7643 (7)	0.060 (2)	
H9	0.1402	−0.0464	0.7399	0.072*	
C10	0.1141 (17)	0.1545 (19)	0.8910 (7)	0.085 (4)	
H10	0.0920	0.1389	0.9566	0.102*	
C11	0.1475 (13)	0.3180 (14)	0.8323 (7)	0.061 (3)	
C12	0.1766 (12)	0.3431 (11)	0.7366 (6)	0.047 (2)	
H12	0.1966	0.4514	0.6963	0.056*	
C13	0.0130 (11)	0.2696 (11)	0.3972 (6)	0.0472 (19)	
H13A	−0.0314	0.3620	0.4230	0.071*	
H13B	−0.0611	0.1702	0.4307	0.071*	
H13C	0.0091	0.2966	0.3310	0.071*	
C14	0.5570 (10)	0.2598 (11)	0.3969 (7)	0.051 (2)	
H14A	0.6137	0.2901	0.3305	0.076*	
H14B	0.6055	0.1568	0.4282	0.076*	
H14C	0.5794	0.3480	0.4249	0.076*	
Br1	0.3949 (2)	−0.25908 (15)	0.00921 (7)	0.0939 (5)	
Br2	0.1448 (2)	0.5021 (2)	0.88551 (10)	0.1127 (6)	

### Atomic displacement parameters (Å^2^)

**Table:**

	*U*^11^	*U*^22^	*U*^33^	*U*^12^	*U*^13^	*U*^23^
Sn1	0.0359 (3)	0.0229 (3)	0.0347 (3)	0.00097 (18)	−0.0081 (2)	−0.0057 (2)
N1	0.051 (4)	0.022 (3)	0.042 (4)	−0.001 (3)	−0.013 (3)	−0.005 (3)
N2	0.132 (9)	0.071 (6)	0.042 (5)	−0.018 (6)	−0.022 (5)	0.008 (5)
O1	0.065 (4)	0.026 (3)	0.038 (3)	0.000 (2)	−0.008 (3)	−0.008 (2)
O2	0.083 (5)	0.021 (3)	0.047 (3)	0.000 (3)	−0.011 (3)	−0.004 (2)
O3	0.069 (4)	0.026 (3)	0.045 (3)	0.000 (2)	−0.013 (3)	−0.006 (2)
O4	0.053 (3)	0.030 (3)	0.041 (3)	0.003 (2)	−0.012 (2)	−0.008 (2)
C1	0.049 (5)	0.025 (4)	0.042 (5)	0.001 (3)	−0.006 (3)	−0.009 (3)
C2	0.047 (4)	0.027 (4)	0.036 (4)	−0.004 (3)	−0.013 (3)	−0.009 (3)
C3	0.047 (4)	0.028 (4)	0.037 (4)	0.005 (3)	−0.011 (3)	−0.009 (3)
C4	0.065 (6)	0.031 (4)	0.050 (5)	−0.003 (4)	−0.016 (4)	−0.013 (4)
C5	0.068 (6)	0.038 (4)	0.036 (4)	−0.008 (4)	−0.011 (4)	−0.011 (4)
C6	0.074 (6)	0.028 (4)	0.043 (5)	−0.001 (4)	−0.016 (4)	−0.006 (3)
C7	0.048 (5)	0.029 (4)	0.042 (4)	0.000 (3)	−0.008 (3)	−0.012 (3)
C8	0.041 (4)	0.043 (4)	0.031 (4)	0.002 (3)	−0.006 (3)	−0.009 (3)
C9	0.080 (7)	0.052 (5)	0.044 (5)	−0.003 (5)	−0.015 (5)	−0.003 (4)
C10	0.091 (9)	0.129 (12)	0.025 (5)	−0.014 (7)	−0.015 (5)	0.000 (6)
C11	0.058 (6)	0.075 (7)	0.049 (6)	−0.018 (5)	−0.003 (4)	−0.022 (5)
C12	0.058 (5)	0.048 (5)	0.034 (4)	0.003 (4)	−0.004 (4)	−0.013 (4)
C13	0.042 (5)	0.046 (5)	0.054 (5)	−0.001 (3)	−0.010 (4)	−0.013 (4)
C14	0.029 (4)	0.049 (5)	0.076 (6)	0.005 (3)	−0.010 (4)	−0.019 (4)
Br1	0.1824 (15)	0.0638 (7)	0.0358 (6)	−0.0216 (8)	−0.0196 (7)	−0.0141 (5)
Br2	0.1363 (13)	0.1431 (14)	0.0718 (9)	−0.0287 (10)	0.0096 (8)	−0.0741 (10)

### Geometric parameters (Å, °)

**Table:**

Sn1—C13	2.089 (8)	C4—C5	1.380 (11)
Sn1—C14	2.086 (8)	C4—H4	0.9300
Sn1—O1	2.189 (5)	C5—C6	1.387 (11)
Sn1—O2	2.546 (6)	C5—Br1	1.873 (8)
Sn1—O3	2.482 (6)	C6—H6	0.9300
Sn1—O4	2.175 (5)	C7—C8	1.499 (11)
Sn1—N1^i^	2.710 (6)	C8—C12	1.376 (11)
N1—C4	1.319 (11)	C8—C9	1.388 (12)
N1—C3	1.327 (9)	C9—H9	0.9300
N1—Sn1^ii^	2.710 (6)	C10—C11	1.394 (16)
N2—C10	1.317 (16)	C10—H10	0.9300
N2—C9	1.319 (13)	C11—C12	1.356 (12)
O1—C1	1.251 (9)	C11—Br2	1.878 (10)
O2—C1	1.250 (9)	C12—H12	0.9300
O3—C7	1.233 (9)	C13—H13A	0.9600
O4—C7	1.272 (9)	C13—H13B	0.9600
C1—C2	1.493 (10)	C13—H13C	0.9600
C2—C6	1.370 (11)	C14—H14A	0.9600
C2—C3	1.403 (10)	C14—H14B	0.9600
C3—H3	0.9300	C14—H14C	0.9600
			
C13—Sn1—O1	97.0 (3)	C5—C4—H4	118.9
C13—Sn1—O2	88.1 (3)	C4—C5—C6	118.7 (8)
C13—Sn1—O3	90.5 (3)	C4—C5—Br1	119.8 (6)
C13—Sn1—O4	97.5 (3)	C6—C5—Br1	121.5 (6)
C14—Sn1—O1	96.4 (3)	C2—C6—C5	119.9 (8)
C14—Sn1—O2	89.2 (3)	C2—C6—H6	120.1
C14—Sn1—O3	88.3 (3)	C5—C6—H6	120.1
C13—Sn1—C14	161.4 (4)	O3—C7—O4	121.8 (7)
C14—Sn1—O4	97.1 (3)	O3—C7—C8	119.9 (7)
O1—Sn1—O2	54.55 (18)	O4—C7—C8	118.3 (7)
O1—Sn1—O4	81.98 (19)	O3—C7—Sn1	67.9 (4)
O4—Sn1—O3	55.59 (18)	O4—C7—Sn1	53.9 (4)
O1—Sn1—O3	137.54 (19)	C8—C7—Sn1	172.1 (5)
O4—Sn1—O2	136.52 (18)	C12—C8—C9	118.2 (8)
O3—Sn1—O2	167.88 (17)	C12—C8—C7	119.6 (7)
C13—Sn1—N1^i^	80.1 (3)	C9—C8—C7	122.2 (8)
C14—Sn1—N1^i^	81.3 (3)	N2—C9—C8	123.7 (9)
O1—Sn1—N1^i^	138.4 (2)	N2—C9—H9	118.2
O2—Sn1—N1^i^	83.81 (18)	C8—C9—H9	118.2
O3—Sn1—N1^i^	84.09 (19)	N2—C10—C11	122.7 (9)
O4—Sn1—N1^i^	139.66 (19)	N2—C10—H10	118.6
C4—N1—C3	119.2 (6)	C11—C10—H10	118.6
C4—N1—Sn1^ii^	119.2 (5)	C12—C11—C10	119.4 (10)
C3—N1—Sn1^ii^	121.6 (5)	C12—C11—Br2	120.3 (8)
C10—N2—C9	117.5 (9)	C10—C11—Br2	120.3 (8)
C1—O1—Sn1	99.8 (4)	C11—C12—C8	118.5 (8)
C1—O2—Sn1	83.2 (5)	C11—C12—H12	120.7
C7—O3—Sn1	84.7 (5)	C8—C12—H12	120.7
C7—O4—Sn1	97.9 (5)	Sn1—C13—H13A	109.5
O2—C1—O1	122.5 (7)	Sn1—C13—H13B	109.5
O2—C1—C2	119.9 (7)	H13A—C13—H13B	109.5
O1—C1—C2	117.6 (7)	Sn1—C13—H13C	109.5
C6—C2—C3	117.2 (7)	H13A—C13—H13C	109.5
C6—C2—C1	121.0 (7)	H13B—C13—H13C	109.5
C3—C2—C1	121.8 (7)	Sn1—C14—H14A	109.5
N1—C3—C2	122.8 (7)	Sn1—C14—H14B	109.5
N1—C3—H3	118.6	H14A—C14—H14B	109.5
C2—C3—H3	118.6	Sn1—C14—H14C	109.5
N1—C4—C5	122.2 (7)	H14A—C14—H14C	109.5
N1—C4—H4	118.9	H14B—C14—H14C	109.5
			
C14—Sn1—O1—C1	−84.8 (5)	C4—C5—C6—C2	0.7 (14)
C13—Sn1—O1—C1	82.3 (5)	Br1—C5—C6—C2	179.5 (6)
O4—Sn1—O1—C1	178.9 (5)	Sn1—O3—C7—O4	−1.2 (8)
O3—Sn1—O1—C1	−179.2 (4)	Sn1—O3—C7—C8	178.9 (7)
O2—Sn1—O1—C1	−0.4 (5)	Sn1—O4—C7—O3	1.4 (9)
C7—Sn1—O1—C1	−180.0 (5)	Sn1—O4—C7—C8	−178.7 (6)
C14—Sn1—O2—C1	98.8 (5)	C14—Sn1—C7—O3	81.1 (5)
C13—Sn1—O2—C1	−99.6 (5)	C13—Sn1—C7—O3	−81.8 (5)
O4—Sn1—O2—C1	−0.7 (6)	O4—Sn1—C7—O3	−178.7 (8)
O1—Sn1—O2—C1	0.4 (5)	O1—Sn1—C7—O3	178.9 (5)
O3—Sn1—O2—C1	176.7 (8)	O2—Sn1—C7—O3	177.7 (7)
C7—Sn1—O2—C1	1.7 (11)	C14—Sn1—C7—O4	−100.1 (5)
C14—Sn1—O3—C7	−99.1 (5)	C13—Sn1—C7—O4	96.9 (5)
C13—Sn1—O3—C7	99.5 (5)	O1—Sn1—C7—O4	−2.4 (5)
O4—Sn1—O3—C7	0.7 (5)	O3—Sn1—C7—O4	178.7 (8)
O1—Sn1—O3—C7	−1.5 (6)	O2—Sn1—C7—O4	−3.6 (11)
O2—Sn1—O3—C7	−177.1 (8)	C14—Sn1—C7—C8	−92 (4)
C14—Sn1—O4—C7	82.3 (5)	C13—Sn1—C7—C8	105 (4)
C13—Sn1—O4—C7	−86.2 (5)	O4—Sn1—C7—C8	8(4)
O1—Sn1—O4—C7	177.8 (5)	O1—Sn1—C7—C8	6(4)
O3—Sn1—O4—C7	−0.7 (4)	O3—Sn1—C7—C8	−173 (4)
O2—Sn1—O4—C7	178.6 (4)	O2—Sn1—C7—C8	5(4)
Sn1—O2—C1—O1	−0.6 (8)	O3—C7—C8—C12	−3.6 (12)
Sn1—O2—C1—C2	178.6 (7)	O4—C7—C8—C12	176.5 (7)
Sn1—O1—C1—O2	0.7 (9)	Sn1—C7—C8—C12	169 (4)
Sn1—O1—C1—C2	−178.6 (6)	O3—C7—C8—C9	176.6 (8)
O2—C1—C2—C6	−3.4 (12)	O4—C7—C8—C9	−3.3 (12)
O1—C1—C2—C6	175.9 (8)	Sn1—C7—C8—C9	−11 (4)
O2—C1—C2—C3	178.6 (7)	C10—N2—C9—C8	0.6 (18)
O1—C1—C2—C3	−2.1 (12)	C12—C8—C9—N2	−0.8 (15)
C4—N1—C3—C2	−0.3 (12)	C7—C8—C9—N2	179.1 (10)
C6—C2—C3—N1	0.9 (12)	C9—N2—C10—C11	−0.8 (19)
C1—C2—C3—N1	179.0 (7)	N2—C10—C11—C12	1.2 (18)
C3—N1—C4—C5	−0.1 (13)	N2—C10—C11—Br2	179.3 (10)
N1—C4—C5—C6	−0.1 (14)	C10—C11—C12—C8	−1.4 (15)
N1—C4—C5—Br1	−178.9 (6)	Br2—C11—C12—C8	−179.4 (7)
C3—C2—C6—C5	−1.1 (13)	C9—C8—C12—C11	1.2 (13)
C1—C2—C6—C5	−179.2 (8)	C7—C8—C12—C11	−178.7 (8)

Symmetry codes: (i) *x*, *y*+1, *z*; (ii) *x*, *y*−1, *z*.
